# Multimodal Approach to Predict Neurological Outcome after Cardiac Arrest: A Single-Center Experience

**DOI:** 10.3390/brainsci11070888

**Published:** 2021-07-01

**Authors:** Lorenzo Peluso, Thomas Boisdenghien, Laila Attanasio, Filippo Annoni, Lili Mateus Sanabria, Paolo Severgnini, Benjamin Legros, Elisa Gouvêa Bogossian, Jean-Louis Vincent, Jacques Creteur, Mauro Oddo, Nicolas Gaspard, Fabio Silvio Taccone

**Affiliations:** 1 Department of Intensive Care, Hopital Erasme, Université Libre de Bruxelles, 1070 Brussels, Belgium; thomas.boisdenghien@ulb.ac.be (T.B.); laila.attanasio88@gmail.com (L.A.); filippo.annoni@erasme.ulb.ac.be (F.A.); lilimateussanabria@gmail.com (L.M.S.); elisagobog@gmail.com (E.G.B.); jlvincent@intensive.org (J.-L.V.); jacques.creteur@erasme.ulb.ac.be (J.C.); ftaccone@ulb.ac.be (F.S.T.); 2 Department of Biotechnology and Life Science, University of Insubria, Cardiac Anesthesiology and Intensive Care, ASST Sette Laghi, 21100 Varese, Italy; paolo.severgnini@uninsubria.it; 3 Department of Neurology, Hopital Erasme, Université Libre de Bruxelles, 1070 Brussels, Belgium; blegros@ulb.ac.be (B.L.); Nicolas.Gaspard@erasme.ulb.ac.be (N.G.); 4 Department of Intensive Care Medicine, Centre Hospitalier Universitaire Vaudois (CHUV), University of Lausanne, 1011 Lausanne, Switzerland; mauro.oddo@chuv.ch

**Keywords:** electroencephalography, post-anoxic, pupillometry, evoked potentials, neuroprognostication, NSE

## Abstract

*Introduction:* The aims of this study were to assess the concordance of different tools and to describe the accuracy of a multimodal approach to predict unfavorable neurological outcome (UO) in cardiac arrest patients. *Methods:* Retrospective study of adult (>18 years) cardiac arrest patients who underwent multimodal monitoring; UO was defined as cerebral performance category 3–5 at 3 months. Predictors of UO were neurological pupillary index (NPi) ≤ 2 at 24 h; highly malignant patterns on EEG (HMp) within 48 h; bilateral absence of N20 waves on somato-sensory evoked potentials; and neuron-specific enolase (NSE) > 75 μg/L. Time-dependent decisional tree (i.e., NPi on day 1; HMp on day 1–2; absent N20 on day 2–3; highest NSE) and classification and regression tree (CART) analysis were used to assess the prediction of UO. *Results:* Of 137 patients, 104 (73%) had UO. Abnormal NPi, HMp on day 1 or 2, the bilateral absence of N20 or NSE >75 mcg/L had a specificity of 100% to predict UO. The presence of abnormal NPi was highly concordant with HMp and high NSE, and absence of N20 or high NSE with HMp. However, HMp had weak to moderate concordance with other predictors. The time-dependent decisional tree approach identified 73/103 patients (70%) with UO, showing a sensitivity of 71% and a specificity of 100%. Using the CART approach, HMp on EEG was the only variable significantly associated with UO. *Conclusions:* This study suggests that patients with UO had often at least two predictors of UO, except for HMp. A multimodal time-dependent approach may be helpful in the prediction of UO after CA. EEG should be included in all multimodal prognostic models.

## 1. Introduction

Sudden cardiac arrest (CA) is a common disease [1]; despite all the advances in cardiopulmonary resuscitation (CPR), survival of CA victims is around 10% [2,3]. After the return of spontaneous circulation (ROSC), the global ischemia/reperfusion injury, (i.e., post cardiac arrest syndrome) including brain injury, myocardial dysfunction, systemic inflammatory response [4], is responsible for a large proportion of in-hospital mortality [5]. Moreover, most deaths will occur because of severe post-anoxic brain injury [6].

Severe post-anoxic brain injury is characterized by persistent coma or even associated with the absence of brainstem reflexes. The use of targeted temperature management (TTM) as a neuroprotective strategy [7] requires the need for sedative and analgesic drugs, which would limit the accuracy of clinical neurological examination to predict neurological outcomes in CA patients [8]. As such, additional prognostic tools are needed. In 2021, International Guidelines recommended assessing neurological prognosis among comatose CA survivors using a combination of different tests; in particular, poor outcome would likely occur when two or more predictors, including highly malignant electroencephalography (EEG, considered as suppressed background or burst-suppression) at >24 h, neuron-specific enolase (NSE) >60 mcg/L at 48 h and/or 72 h, absence of pupillary and/or corneal reflex at >72 h, absence of cortical response (N20) to short-latency somatosensory evoked potentials (SSEPs) at >24 h, onset of status myoclonus ≤72 h, extensive brain injury at MRI/CT scan, were present [3].

Quantifying post-anoxic brain injury is essential to avoid inappropriate intensive care for patients with irreversible damage and to delay the awakening for patients with a chance of recovery. In recent years, several studies have provided new insights into the prognostic values of all these tools, in particular early EEG findings and pupillary assessment using automated pupillometry [9,10,11,12]. Moreover, it remains unclear which is the optimal combination of prognostic tools, as some could provide the same information and therefore be considered as redundant. Finally, as the different prognostic tools have the best predictive value at different time-points, the evaluation of a combination of different prognostic tools based on a multimodal decision would provide accurate information on how to predict UO in this setting.

The aims of this study were therefore to: (a) evaluate the prognostic value of different predictive tools in CA patients; (b) assess the concordance of different tools to identify UO; and (c) describe the accuracy of a multimodal approach to predict neurological prognosis.

## 2. Materials and Methods

### 2.1. Study Design

This study was a monocentric retrospective study using data prospectively collected that was performed between January 2016 and March 2019. The study was approved by the Ethics Committee of Erasme Hospital (reference: P2019/211), which waived the need for an informed consent because its retrospective design.

### 2.2. Patients

This cohort study included adult patients (>18 years) who remained with a Glasgow coma scale (GCS) <9 after hospital admission and were admitted into the intensive care unit (ICU) of Erasme Hospital. We excluded patients with early deaths or awakening (<24 h) who did not have at least two prognostic tools assessed. Patients’ management is described elsewhere [13]. Unfavorable neurological outcome at 3 months was defined as cerebral performance category score (CPC) 3–5.

### 2.3. Neurological Outcome Assessment

Neurological examination (at minimum, motor response and PLR using a manual flash lamp) was performed on admission and then at least twice daily thereafter. “Poor motor response” was defined as absent motor response or posturing (GCS-M < 3) on day 3. EEG recording was initiated as soon as possible after ICU admission and continued for at least 48 h; the presence at EEG of highly malignant patterns (HMp, considered as persistent suppression, suppression-burst and its variants suppression-seizure and suppression-generalized periodic discharges/generalized spike and wave) and lack of reactivity, considered as absence of EEG changes to external stimulation were recorded during the first 24 h (day 1) and at day 2. For this study, all EEG traces were reviewed by an experienced neurophysiologist, who was blinded to the neurological status of the patient. Repeated pupillary assessment using an automated pupillometry (Neuroptics, Irvine, CA, USA) was performed every 6 h; the neurological pupil index (NPi) was recorded and the worst measurement on day 1 and day 2 was collected; an NPi ≤ 2 was defined as “abnormal” [11]. The highest NSE values over the first 3 days was collected; high NSE was defined for values above 75 mcg/L [14]. The results of SSEPs and timing of SSEPs assessment were also recorded, whenever available.

Life-support therapies were maintained for at least 72 h after CA; the decision process for withdrawal of life-support therapies was interdisciplinary and based on the existing guidelines at the moment of the study, in combination with clinical evaluation (i.e., status myoclonus) and EEG findings (i.e., presence of non-convulsive status epilepticus refractory to three anti-epileptic drugs and continuous sedation).

### 2.4. Data Collection

We collected demographic characteristics, data on CA (i.e., location, initial rhythm, cause of CA, bystander CPR, time to ROSC, drugs administered), lactate and creatinine on admission, as well as the use of different interventions (i.e., vasopressors, TTM, renal replacement therapy) during the ICU stay. Shock was defined as the use of vasopressors for more than 6 consecutive hours during the first 2 days after admission. 

### 2.5. Statistical Methods

Data were tested for normality using the Kolmogorov–Smirnov test and were presented as median (interquartile range) or mean ± standard deviation, as appropriate. Categorical variables are presented as counts (%). Categorical variables were compared using the Fisher exact test or Chi-square test, as appropriate, and Student t-test or Mann–Whitney U test was used to compare continuous variables, as appropriate. We analyzed the distribution of different prognostic tools in patients with FO and UO; sensitivity, specificity, positive and negative predictive values (PPV and NPV) for UO were calculated for all of the different tools. False positive rate (FPR) for each tool was calculated as: false positive/FO. Concordance between the different tools to predict UO was defined as the presence of two or more tools suggesting UO and expressed as percentage. “High” concordance between two or more predictors was arbitrarily defined if >75%; “moderate” concordance was defined if 50–74% and “weak” concordance if <50%. 

To assess the best multimodal approach, we first used a time-dependent decisional tree (i.e., NPi on day 1; HMp on day 1–2; absent N20 on day 2–3; highest NSE value over the first 3 days); this approach was based on the time of assessment, i.e., the first tool being NPI on day 1, followed by HMp on day 1 or 2, absence of N20 on day 2–3 and, finally, the highest NSE value within the first 72 h. Thereafter, we analyzed the most significant predictors associated with UO by classification and regression tree (CART) analysis, which allows partitions observations in a matched data set, consisting of a categorical dependent variable (i.e., UO) and one or more independent explanatory variables (i.e., predictors), into progressively smaller groups. Each potential binary splits are examined and the split that maximizes the discrimination of the two resulting groups (i.e., UO vs. others) is chosen. The analysis is therefore continued into the remaining patients to reassess the possibility to maximize discrimination again [15]. A *p* value < 0.05 was considered statistically significant. Statistical analyses were performed using SPSS (IBM SPSS Statistics 25.0 for Macintosh).

## 3. Results

### 3.1. Study Population

During the study period, 175 patients were admitted after a CA; of those, 8 died within a few hours after admission and 30 patients had incomplete data on multimodal monitoring. A total of 137 patients (median age 65; male 95/137) was then included in the final analysis; 103 (75%) of those had UO. Most of the patients had an OHCA (72%); median time to ROSC was 21 min and 37% of patients had an initial shockable rhythm (Table 1). Patients with UO were older, less frequently had a witnessed CA and initial shockable rhythm but had a longer time to ROSC, more often a non-cardiac origin of the arrest, received higher dose of epinephrine during CPR, and presented with higher lactate levels on admission than those with FO (Table 1).

### 3.2. Prediction of Unfavorable Outcome for Each Predictor 

NPi was available for all patients; EEG findings were also available for all patients (128, 90% on day 1 and 111, 78% on day 2), NSE values were measured in 113 (83%) patients and SSEPs were performed in 60 (44%) patients. A total of 30 patients presented abnormal NPi and all of them had UO; the sensitivity was 29% with a specificity of 100%, with a FPR or 0%. The presence of HMp on day 1 and 2 was observed in 60/128 (47%) and 19/111 (17%) patients, respectively; all of them presented UO (FPR of 0% to predict UO). The bilateral absence of N20 was observed in 24 patients; all of them presented UO, resulting in a sensitivity of 45% and a specificity of 100, with a FPR of 0%. High NSE values was observed in 38 patients, all of them presenting UO; resulting in a sensitivity of 45% and a specificity of 100%, with a FPR of 0% (Table 2).

Among others clinical predictors, bilateral absence of PLR at day 3 was observed in 26 patients, 21 of them presented UO, showing a sensitivity of 20%, a specificity of 85% with a false positive rate of 15%. Poor motor response at day 3 was observed in 114 patients with a FPR of 47% and the presence of myoclonus at any time showed a sensitivity of 27% with a specificity of 97% (FPR 3%).

### 3.3. Concordance of Different Prognostic Tools to Predict Unfavorable Outcome

Concordance among different prognostic tools is shown in Supplemental Table S1. The presence of abnormal NPi at day 1 or 2 was highly concordant with the presence of HMp on EEG. Abnormal NPi on day 2 was also highly concordant with high NSE. The bilateral absence of N20 or high NSE were highly concordant with HMp on EEG on day 1 or 2. Conversely, HMp on EEG on day 1 or 2 had weak to moderate concordance with other predictors. Moderate to weak concordance were observed also for the concordance of at least 3 predictors. Three patients had isolated abnormal NPi on day 1 and 2 patients on day 2; isolated HMp was observed in 16 patients on day 1 and 6 patients on day 2. No patients showed isolated bilaterally absent N20, whereas isolated high NSE was observed in 5 patients.

### 3.4. Time-Dependent Prognostic Model to Predict Unfavorable Outcome

**Figure 1:**
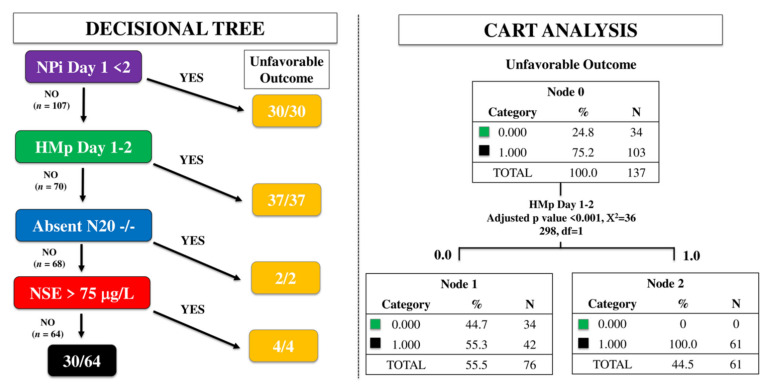
Time-dependent decision tree (left) and classification and regression tree (CART) analysis (right) to assess the prognostic accuracy of a multimodal approach. NPi, neurological pupil index; HMp, highly malignant patterns on the EEG; N20, cortical response on somato-sensory evoked potentials testing; NSE, neuron specific enolase.

Using the time-dependent decisional tree approach, 30/137 patients had abnormal NPi on day 1 and all of them had a UO. Among the 107 remaining patients, 37 (35%) had HMp on EEG on day 1 or 2 and all of them had UO. Of the 70 patients without abnormal NPi and HMp, 2 had bilaterally absent N20 on day 3 and all of them had UO. Of the remaining 68 patients, 4 had high NSE and all of them had UO (Figure 1). As such, this multimodal time-dependent approach identified 73 out of 103 patients (70%) with UO, showing a sensitivity 71%, a specificity 100%, PPV 100% and NPV 53% to predict UO, with a FPR 0%. Using the CART approach, HMp on EEG on day 1 or 2 was the only variable significantly associated with UO (Figure 1).

Among the 64 patients without any of the predictors of UO, 34 had FO and 30 UO; we found no significant differences in proportion of reactive EEG, SSEPs at day 3 and NSE values between these two groups; however, lower NPi values were observed in patients with UO when compared with others (4.2 (3.8–4.4) vs. 4.5 (4.2–4.6); *p* < 0.01—Table 3). 

## 4. Discussion

In this study, we observed that: (a) abnormal NPi, HMp on EEG, bilaterally absent N20 and high NSE had a high specificity and PPV to predict UO in CA patients; (b) the presence of abnormal NPi, absent N20 and high NSE were highly concordant with HMp on EEG, while HMp had moderate concordance with other predictors; (c) only a minority of patients had several concomitantly present predictors of UO, but isolated HMp or absent N20 was observed in some patients; (d) a time-dependent multimodal approach identified 70% of patients with UO, showing a specificity 100% and a FPR of 0%, while the CART analysis identified HMp on EEG at the best predictor for UO. 

Since 2014, recommendations on prognostication of post-anoxic brain injury are based on the European Society of Intensive Care Medicine (ESICM) and the European Resuscitation Council (ERC) guidelines, which were founded on a systematic review of literature and grading of the existing evidence [28]. Despite some relevant statements, these guidelines also presented several limitations: (a) no early prognostic tool was indicated, while some findings are extremely relevant when identified in the first 24 h after arrest (i.e., HMp on EEG) [14]; (b) no specific indications for the selection of specific tools for the multimodal approach was proposed; (c) significant publications have been released in this field in the last years which have resulted in some outdated statements. A more recent systematic review and revision of these guidelines also identified clinical, biochemical, neurophysiological, and radiological tests that have a potential to predict UO with no false-positive predictions within the first week after CA [29]. 

In particular, the role of EEG as a predictor of neurological outcome after CA has been evaluated in several publications and should probably be reconsidered [30,31,32]. The main findings on EEG are: (a) the use of a standardized nomenclature can avoid overestimation of highly malignant patterns (i.e., discontinuous EEG classified as BS) [16,17]; (b) EEG recordings suggesting UO are more frequent in the 12–24 h after ICU admission [31]; (c) in case of burst-suppression background, sedation with propofol might be a significant limitation on the accuracy of EEG [18]; (d) post-anoxic seizures might be associated with neurological recovery if associated with an early continuous EEG background and prompt therapy [19]; (e) EEG findings may identify patients with post-anoxic myoclonus and without potential recovery [20]. In our study, we focused on HMp as the most robust predictor of UO in CA patients [29]. We reported three interesting findings: (a) HMp may occur in the absence of other predictors of UO (i.e., weak to moderate concordance); (b) some patients with UO could be identified using only HMp on EEG; (c) HMp had the highest predictive value among all other predictors in the CART analysis. As such, in a multimodal approach, EEG should always be included into the prognostic algorithm. Moreover, in settings of limited available prognostic tools, EEG should probably be the one to implement. We observed a lower accuracy to predict outcome for EEG reactivity, which might be due to the relatively low inter-rater agreement, the lack of standardized test to assess it [16] and to the confounder effects of concomitant sedation.

Concerning other tools, a NPi ≤2 was associated with a PPV of 100% already at 24 h after arrest to predict UO [11]. Moreover, NPi was more accurate than standard or quantitative PLR to predict UO. Additionally, two recent studies showed that a cut-off of NSE >60 mcg/L and 80 mcg/L was associated with a FPR of 2% [10,20]; we used a cut-off of 75 mcg/L, which has been proposed as an accurate threshold to predict UO in previous studies [21,22]. Although isolated measurements have some limitations, NSE correlated well with other parameters, suggesting extended post-anoxic brain injury and can better quantify as a continuous variable (i.e., rather than a dichotomous variable) the severity of brain injury in CA patients.

Few studies have evaluated the role of a multimodal approach to predict neurological outcome after CA. In one study, the combination of SSEPs, cerebral CT-scan, and EEG increased the sensitivity of UO prediction from 30–54% to 61% [23]. In another study, a prognostic model including brain CT-scan, NSE, EEG, SSEPs, and PLR predicted UO with a 0% FPR [24], significantly higher than each prognostic modality alone. A combination of clinical examination, EEG reactivity, and NSE yielded the best predictive accuracy to predict UO in this setting [22]. Finally, Youn et al. reported combining brain CT-scan with clinical examination and EEG was superior to any individual test for predicting mortality and neurologic outcome [32]. However, the addition of high NSE cut-offs and accurate EEG classification had minor effects on the sensitivity of a multimodal approach including PLR and SSEPs, as suggested by 2015 Guidelines [25]. Although the CART analysis suggests that EEG would be sufficient to adequately predict neurological outcome in these patients, the combination of several parameters would provide more robustness to the prognostication process and should not be discarded.

Very limited data are available on the concordance of several predictors in this setting. In one study focusing on NSE, all patients with NSE >33 mcg/L had extensive brain injury on magnetic resonance imaging and most of them also lacked cortical responses on SSEP bilaterally; however, NSE poorly correlated with EEG patterns [26]. In a second study, NSE values and EEG findings were strongly correlated; also, median NSE peak values were higher in patients with unreactive background and discontinuous patterns than others [21]. From our findings, several patients had at least two concomitant predictors of UO; although this could be considered as a “redundant” information, physicians would be more comfortable to consider life-sustaining therapies if two or more predictors of UO are present, as this suggests an extensive hypoxic brain damage. Moreover, if a comatose CA patient undergoing a multimodal prognostic approach would present isolated abnormal NPi, high NSE or bilaterally absent N20, which was a very rare finding in our cohort, the physician might consider repeating some of these examinations to exclude technical issues or sampling inaccuracy. However, discordance between predictive tools should not be considered as a sign of imprecision or inaccuracy, as they evaluate different anatomical areas of the brain (i.e., NPi, the brainstem; SSEPs, the spinothalamic tract; NSE, the neuronal damage; EEG, the presence of cortical ischemia), which have different sensitivity to the anoxic injury.

Our study had several limitations. First, this was a retrospective study. Second, the study was single-center and local practices on limitation of life-sustaining therapies might limit their generalizability to other ICUs. Third, the risk of self-fulfilling prophecy could have influenced some results, as clinicians were unblinded to results of the prognostic tests. Fourth, as this study focused only on comatose patients requiring the whole prognostication model, around 20% of patients were excluded. Fifth, all predictors were not available for all patients and this has limited the possibility to test different combinations to determine the optimal prognostic algorithm in this setting, in particular because data was missing due to random factors (i.e., lack of reported data) and lack of necessity (i.e., patients who regained consciousness), which may have led to significant bias. Sixth, we did not include brain imaging (i.e., CT-scan or magnetic resonance imaging, MRI) in this multimodal approach. Finally, the number of patients with favorable neurological outcome was quite limited and further analyses on this issue, as reported in another study [27], were not performed.

## 5. Conclusions

This study suggests that patients with UO had often at least two predictors of UO; isolated predictors are observed mainly with EEG. A multimodal time-dependent approach, including NPi, HMp, SSEPs, and NSE, may be helpful in the prediction of UO after CA. EEG should be included in all multimodal prognostic models.

## Figures and Tables

**Table 1:** Clinical characteristics of the study population according to the neurological outcome (FO = favourable; UO = unfavourable).

	All Patients (*n* = 137)	FO (*n* = 34)	UO (*n* = 103)	*p* Value
Male gender, *n* (%)	95 (69)	26 (77)	69 (67)	0.39
Age, years	65 (54–72)	60 (50–67)	67 (55–73)	<0.01
**CARDIAC ARREST**				
Witnessed, *n* (%)	116 (85)	33 (97)	83 (81)	0.03
Bystander CPR, *n* (%)	84 (61)	23 (68)	61 (59)	0.42
Out-of-Hospital Cardiac arrest, *n* (%)	98 (72)	28 (82)	70 (68)	0.13
Time to ROSC, min	21 (15–32)	15 (10–21)	25 (18–35)	<0.01
Epinephrine, mg	3 (2–6)	2 (1–5)	4 (2–7)	<0.01
Non-cardiac Origin, *n* (%)	68 (50)	8 (24)	60 (58)	<0.01
Shockable Rhythm, *n* (%)	50 (37)	21 (62)	29 (28)	<0.01
**COMORBID DISEASES**				
Chronic Heart Failure, *n* (%)	21 (15)	6 (18)	15 (15)	0.78
Hypertension, *n* (%)	53 (39)	11 (32)	42 (41)	0.42
Coronary Artery Disease, *n* (%)	43 (31)	14 (41)	29 (28)	0.20
Diabetes, *n* (%)	33 (24)	7 (21)	26 (25)	0.65
COPD/Asthma, *n* (%)	23 (17)	6 (18)	17 (17)	0.99
Previous neurological disease, *n* (%)	13 (10)	3 (9)	10 (10)	0.99
Chronic Renal Failure, *n* (%)	10 (7)	2 (6)	8 (8)	0.99
Liver Cirrhosis, *n* (%)	3 (2)	0	3 (3)	0.57
Others immunosuppressive agents, *n* (%)	2 (2)	0	2 (2)	0.99
**DURING ICU STAY**				
Arterial Lactate on admission (mEq/L)	6.8 (4.4–9.4)	4.8 (3.5–6.5)	7.3 (5.1–10.7)	<0.01
Creatinine on admission (mg/dL)	1.3 (1.0–1.7)	1.2 (0.9–1.4)	1.3 (1.1–1.8)	0.04
TTM, *n* (%)	116 (85)	29 (85)	87 (84)	0.99
MV, *n* (%)	137 (100)	34 (100)	103 (100)	1.00
RRT, *n* (%)	16 (12)	3 (9)	13 (13)	0.76
Vasopressor any time, *n* (%)	117 (85)	27 (79)	90 (87)	0.27
Dobutamine any time, *n* (%)	71 (52)	15 (44)	56 (54)	0.33
Shock, *n* (%)	62 (45)	12 (35)	50 (49)	0.23
Corticosteroids, *n* (%)	21 (15)	3 (9)	18 (18)	0.28
IABP, *n* (%)	4 (3)	2 (6)	2 (2)	0.26
ECMO, *n* (%)	18 (13)	4 (12)	14 (14)	1.00
**OUTCOMES**				
ICU Stay, days	4 (3–8)	10 (6–13)	4 (2–5)	<0.01
ICU Mortality, *n* (%)	96 (70)	-	96 (93)	<0.001

ICU, intensive care unit; CPR, cardiopulmonary resuscitation; ROSC, return to spontaneous circulation; IABP, iIntra-aortic balloon pump; ECMO, extra corporeal membrane oxygenation; COPD, chronic obstructive pulmonary disease; MV, mechanical ventilation; TTM, targeted temperature management; RRT, renal replacement therapy.

**Table 2:** Different prognostic tools according to neurological outcome (FO, favourable; UO, unfavourable).

	FO (*n* = 34)	UO (*n* = 103)	*p* Value	Sens Spec	PPV NPV	FPR
**CLINICAL**						
Bilateral Absence of PLR Day 3, *n* (%)	5 (15)	21 (20)	0.62	20% 85%	81% 26%	15%
Poor Motor Response Day 3, *n* (%)	16 (47)	98 (95)	<0.01	95% 53%	86% 78%	47%
Myoclonus Any Time, *n* (%)	1 (3)	28 (27)	<0.01	27% 97%	97% 31%	3%
**AUTOMATED PUPILLOMETRY**						
NPi Day 1	4.5 (4.2–4.6)	3.9 (0–4.4)	<0.01			
NPi <2 Day 1, *n* (%)	0	30 (29)	<0.01	29% 100%	100% 32%	0%
NPI Day 2	4.6 (4.2–4.7)	4.1 (0–4.5)	<0.01			
NPi <2 Day 2, *n* (%)	0	26 (25)	<0.01	25% 100%	100% 31%	0%
**EEG**						
HMp Day 1, *n* (%)	0	60/96 (63)	<0.01	63% 100%	100% 47%	0%
HMp Day 2, *n* (%)	0	19/82 (23)	<0.01	23% 100%	100% 32%	0%
Unreactive EEG Day 1, *n* (%)	9/32 (28)	76/96 (79)	<0.01	79% 72%	89% 53%	28%
Unreactive EEG Day 2, *n* (%)	3/29 (10)	48/82 (59)	<0.01	59% 90%	94% 43%	10%
**OTHERS**						
Bilateral Absence of N20 Day 3, *n* (%)	0	24/53 (45)	0.04	45% 100%	100% 19%	0%
NSE value > 75 μg/L	0	38/85 (45)	<0.01	45% 100%	100% 37%	0%
Highest NSE value over 3 days, mcg/L	26 (21–38)	59 (33–141)	<0.01			

PLR, pupillary light reflex; NPi, neurological pupillary index; NSE, neuron-specific enolase; EEG, electroencephalography; HM, highly malignant patterns; Sens, sensitivity; Spec, specificity; PPV, positive predictive value; NPV, negative predictive value; FPR, false positive rate.

**Table 3:** Characteristics of patients (*n* = 64) without predictors of unfavorable neurological outcome (i.e., abnormal NPi, highly malignant EEG patterns, high NSE and bilaterally absent N20). Data are presented as count (%) or median [IQRs].

	FO *n* = 34	UO *n* = 30
**NPi on day 1**		
*Total of measurements, n* *Median [IQR]*	34 4.5 [4.2–4.6]	30 4.2 [3.8–4.4] *
**EEG on day 2**		
*Total of measurements, n* *Reactive, n (%)*	29 26 (90)	29 22 (76)
**SSEP on day 2–3**		
*Total of measurements, n (%)* *N20 bilaterally present, n (%)*	7 7 (100)	13 13 (100)
**Highest values of NSE, mg/L**		
*Total of measurements, (%)* *Median [IQR]*	28 26 [16–27,33–38]	25 32 [19–27,33–38]

* *p* < 0.05. Legend: FO, favorable outcome; UO, unfavorable outcome; NPi, neurologic pupil index; EEG, electroencephalography; SSEP, somatosensory evoked potentials.

## Data Availability

The data presented in this study are available on request from the corresponding author.

## Supplementary Materials

The following are available online at https://www.mdpi.com/article/10.3390/brainsci11070888/s1, Table S1: Concordance of different prognostic tools within the multimodal approach.
