# Supplementary material for: Multimodal Approach to Predict Neurological Outcome after Cardiac Arrest: A Single-Center Experience
Source: Brain Sci. 2021 Jul 1;11(7):888. doi: 10.3390/brainsci11070888 (PMC8303816; doi:10.3390/brainsci11070888)
Supplement: Supplementary file 1 [file brainsci-11-00888-s001.zip › brainsci-1274729-supplementary.pdf]

Table S1. Concordance of different prognostic tools within the multimodal approach. Data are shown as counts (%).

	**NPi < 2*** **(n=30)**	**HMp EEG*** **(n=61)**	**SSEPs** **(n=24)**	**NSE** **(n=38)**	**NPi+EEG+SSEPs** **(n=11)**	**NPi+EEG+NSE** **(n=17)**	**NPi+SSEPs+NSE** **(n=9)**	**EEG+SSEPs+NSE** **(n=14)**	**ALL** **(n=8)**	**ALONE**
NPi < 2 Day 1 (n=30)	-	24/30 (80)	11/17 (65)	19/27 (70)	11/17 (65)	17/27 (63)	9/16 (56)	8/16 (50)	8/16 (50)	3/64 (5)
NPi < 2 Day 2 (n=26)	-	21/26 (81)	11/16 (69)	18/23 (78)	11/16 (69)	16/23 (70)	9/15 (60)	8/15 (53)	8/15 (53)	2/67 (3)
HMp EEG Day 1 (n=60)	24/60 (40)	-	20/34 (59)	32/50 (64)	9/34 (26)	17/50 (34)	8/31 (26)	14/31 (45)	8/31 (26)	16/75 (21)
HMp EEG Day 2 (n=19)	6/19 (32)	-	8/14 (58)	9/16 (56)	4/14 (29)	4/16 (25)	3/12 (25)	5/12 (42)	3/12 (25)	6/66 (9)
N20-/- Day 3 (n=24)	11/24 (46)	20/24 (83)	-	15/20 (75)	11/24 (45)	8/20 (40)	9/20 (45)	14/20 (70)	8/20 (40)	0/21 (0)
Highest NSE > 75 mg/L (n=38)	19/38 (50)	32/38 (84)	15/25 (60)	-	9/25 (36)	17/38 (45)	9/25 (36)	14/25 (56)	8/25 (32)	5/57 (7)

* The values of NPi and EEG in the columns are considered as the worst in both day 1 or 2.

NPi = neurologic pupil index; HMp EEG = highly malignant EEG pattern; SSEPs = somatosensory evoked potentials; N20 -/- = bilaterally absence of cortical responses to SSEPs; NSE = neuron-specific enolase.
